# The cross-talk between macrophages and tumor cells as a target for cancer treatment

**DOI:** 10.3389/fonc.2023.1259034

**Published:** 2023-11-14

**Authors:** Muhammad Aizaz, Aakif Khan, Faisal Khan, Maria Khan, Ebraheem Abdu Musad Saleh, Maryum Nisar, Natalia Baran

**Affiliations:** ^1^ Shandong Provincial Key Laboratory of Animal Resistance Biology, College of Life Sciences, Shandong Normal University, Jinan, China; ^2^ Centre of Excellence in Molecular Biology, University of the Punjab, Lahore, Pakistan; ^3^ Center of Biotechnology and Microbiology, University of Peshawar, Peshawar, Pakistan; ^4^ Department of Chemistry, College of Arts & Science, Prince Sattam Bin Abdulaziz University, Alkharj, Saudi Arabia; ^5^ School of Interdisciplinary Engineering & Sciences, National University of Sciences and Technology, Islamabad, Pakistan; ^6^ Department of Leukemia, The University of Texas MD Anderson Cancer Center, Houston, TX, United States

**Keywords:** macrophages, TAMs, tumor cells, cross-talk, hematological malignancies, cancer progression

## Abstract

Macrophages represent an important component of the innate immune system. Under physiological conditions, macrophages, which are essential phagocytes, maintain a proinflammatory response and repair damaged tissue. However, these processes are often impaired upon tumorigenesis, in which tumor-associated macrophages (TAMs) protect and support the growth, proliferation, and invasion of tumor cells and promote suppression of antitumor immunity. TAM abundance is closely associated with poor outcome of cancer, with impediment of chemotherapy effectiveness and ultimately a dismal therapy response and inferior overall survival. Thus, cross-talk between cancer cells and TAMs is an important target for immune checkpoint therapies and metabolic interventions, spurring interest in it as a therapeutic vulnerability for both hematological cancers and solid tumors. Furthermore, targeting of this cross-talk has emerged as a promising strategy for cancer treatment with the antibody against CD47 protein, a critical macrophage checkpoint recognized as the “don’t eat me” signal, as well as other metabolism-focused strategies. Therapies targeting CD47 constitute an important milestone in the advancement of anticancer research and have had promising effects on not only phagocytosis activation but also innate and adaptive immune system activation, effectively counteracting tumor cells’ evasion of therapy as shown in the context of myeloid cancers. Targeting of CD47 signaling is only one of several possibilities to reverse the immunosuppressive and tumor-protective tumor environment with the aim of enhancing the antitumor response. Several preclinical studies identified signaling pathways that regulate the recruitment, polarization, or metabolism of TAMs. In this review, we summarize the current understanding of the role of macrophages in cancer progression and the mechanisms by which they communicate with tumor cells. Additionally, we dissect various therapeutic strategies developed to target macrophage–tumor cell cross-talk, including modulation of macrophage polarization, blockade of signaling pathways, and disruption of physical interactions between leukemia cells and macrophages. Finally, we highlight the challenges associated with tumor hypoxia and acidosis as barriers to effective cancer therapy and discuss opportunities for future research in this field.

## 1 Introduction

The tumor microenvironment (TME), the environment surrounding cancer cells, is crucial to cancer development, providing a stage for several hallmarks of cancer like tumor growth, uncontrolled tumor cell proliferation, evasion of growth suppression, immune system evasion, angiogenesis, tumor migration and invasion, tumor progression, metastasis, or emergence of treatment resistance to occur (1, 2). The TME consists of diverse cellular and extracellular components (3, 4). The cellular compartment of the TME consists of stromal cells, including cancer-associated fibroblasts (CAFs), endothelial cells (ECs), pericytes, and mesenchymal stem cells, as well as diverse immune cells, which typically include tumor-infiltrating lymphocytes, microglia, macrophages, and dendritic cells (DCs) (5, 6). This compartment of the TME can be divided further into two functional subcategories of cells: immune-stimulating cells, which facilitate the anticancer immune response, and immunosuppressive cells, which inhibit the anticancer immune response to promote tumor progression (7). The ongoing interaction between these elements and tumor cells creates a dynamic network that promotes tumorigenesis (5). These interactions among different cell types occur within a unique environment for each cancer type and cancer stage noncellular component of the TME. The non-cellular TME consists of the extracellular matrix (ECM), mainly including structural proteins (e.g., collagen, elastin, and tenascin), glycosaminoglycans (e.g., hyaluronic acid), proteoglycans (e.g., chondroitin sulfate, dermatan sulfate, heparin sulfate, heparan sulfate, and keratan sulfate), matricellular proteins (e.g., osteonectin, osteopontin, and thrombospondin), adhesion proteins (e.g., fibronectin and laminin), and a variety of signaling chemicals (e.g., cytokines, chemokines, and growth factors) (5, 6, 8).

TME composition, both cellular and extracellular, may change depending on the stage of tumor progression and undergoes continuous reorganization via several intrinsic and extrinsic processes (9, 10). The key intrinsic factors influencing the risk of tumor development and progression are genetic alterations, whereas extrinsic contributors to TME remodeling are hypoxia, acidosis, and inflammation, which impact the final composition of both the cellular construction of TME and the extracellular TME matrix (5).

Although the specific composition of a TME may depend on the tissue origin of the tumor, independent of cancer type, increased infiltration of tumor-associated macrophages (TAMs), monocytes, and DCs is common to protumorigenic TMEs (11). Also, protumorigenic TMEs are frequently accompanied by T helper 2 (Th2) cells, myeloid-derived suppressor cells (MDSCs), neutrophils (particularly of type N2), tolerogenic DCs (with immunosuppressive properties, priming the immune system into a tolerogenic state against various antigens, causing clonal T-cell deletion and anergy, suppressing memory and effector T-cell responses, and producing and activating regulatory T cells [Tregs]), and other Tregs (5, 12) as shown in **Table 1**. In comparison, antitumorigenic TMEs are often enriched in CD8^+^ cytotoxic T lymphocytes, Th1 cells, classically activated M1 macrophages, neutrophils, and natural killer (NK) cells.

**Table 1:** The components of antitumorigenic and protumorigenic TMEs [adapted from Hourani et al. (6)].

	TME	
Component	Antitumorigenic	Protumorigenic
Macrophages	M1 (CD86, TLR4)	M2 (CD163, CD206)
Th cells	Th1 cells	Th2 cells
DCs	Mature DCs	Tolerogenic DCs (CD80^low^, CD86^low^)
T cells	Cytotoxic CD8^+^ T cells	Tregs
Other cells	NK cells	MDSCs
Cytokines	IL-2, IL-12, IFN-γ	IL-4, IL-6, IL-10, TGF-β, IFN-γ
Growth/angiogenic factors	GM-CSF	GM-CSF, EGF, HGF, FGF, VEGF
Chemokines	CXCL9, CXCL10	CCL2

Tolerogenic DCs consist of a heterogeneous pool of DCs with immunosuppressive properties that prime the immune system into a tolerogenic state in response to various antigens.

GM-CSF, granulocyte-macrophage colony-stimulating factor; HGF, hepatocyte growth factor.

These differences in cellular tumor composition, particularly in the nature, density, immune functional orientation, and distribution of immune cells within a tumor, became a further basis for identifying immune tumor profiles associated with distinct responses to treatment with immune checkpoint inhibitors and therefore distinct survival and patient outcomes (3, 4, 9, 10). This stratification of patients with solid tumors according to composition of immune environment demonstrated the central role of the immune system in guiding therapeutic decisions and enables one to distinguish four types of tumors: hot, cold, altered-excluded, and altered-immunosuppressed tumors (9, 10, 13, 14).

Hot tumors are attributed to infiltration of TMEs mostly by T cells (15–17). They intensify the immune response, engaging it to recognize and attack tumor cells and produce a good response to immunotherapy, including that with immune checkpoint inhibitors (17). Cold tumors, on the other hand, are characterized by deficient immune cell infiltration in the TME, resulting in evasion of immune detection and responses to immune effector cells via several mechanisms, such as immunosuppressive growth factors and cytokines produced by tumor cells. A hot TME is generally seen as more favorable than cold TME in the context of cancer treatment because it suggests that the immune system is aggressively combating the tumor (15). Furthermore, an altered-excluded tumor is characterized by TME infiltration of CD8^+^T cells located at the edge of the invasive margin of the tumor dominated by an abnormal vasculature (and consequent hypoxia) and a dense stroma, while altered-immunosuppressed tumors are characterized by the presence of a low degree of immune infiltration and an immunosuppressive, often hypoxic TME that limits further recruitment of immune cells and promotes an expansion of tumor (9, 10, 13–17).

Besides differences in the cellular composition of TME, distinctions in cytokines and secreted growth factors can also be found in TME, which help in the identification and characterization (8, 16–19). Most common in the latter milieu are growth factors associated with inflammation, such as granulocyte-macrophage colony-stimulating factor, epidermal growth factor (EGF), hepatocyte growth factor, and fibroblast growth factor (FGF) which are accompanied by vascular endothelial growth factor (VEGF) and stimulate angiogenesis (3, 5, 7, 8, 12, 20, 21). A protumorigenic TME is saturated with several supporting tumor growth cytokines like interleukin (IL)-4, IL-6, and IL-10 as well as transforming growth factor (TGF)-β, interferon (IFN)-γ, and chemokines such as chemokine (C-C motif) ligand 2 (CCL2) (3, 5, 7, 8, 12, 20, 21). Conversely, an antitumorigenic TME is frequently enriched in IL-2 and IL-12 along with IFN-γ, granulocyte macrophage-stimulating factor, and chemokines like C-X-C motif chemokine ligand 9 (CXCL9) and CXCL10 (22). However, the role of specific cell populations and signaling molecules in TME depends on many other factors, such as the presence of programmed death-ligand 1 (PD-L1) receptors that are often upregulated in tumor tissue and, through cooperation with IFN-γ, can induce tumor growth-promoting properties (23–26). Like IFN-γ, granulocyte macrophage-stimulating factor is known to effectively elicit anticancer immune responses, but it can also trigger tumor development and metastasis, demonstrating its context-dependent mechanism of action (27, 28).

Remodeling of the ECM and lymphatic and blood vessels caused by autocrine and paracrine signaling between the TME and cancer cells may control invasion of the cells (29). CAFs and TAMs are the two crucial cell populations impacting and modulating the maturation and modulation of the TME, remodeling of the ECM, and modulation of metabolism and angiogenesis as well as cross-talk between tumor cells and tumor-infiltrating immune cells via the production of growth factors, cytokines, and chemokines (30). Upon interaction with tumor cells, CAFs secrete or shed diverse proteins such as collagens, glycoproteins, and proteoglycans. They can also transmit autocrine and paracrine signals, including cytokines/chemokines, growth factors, mRNAs, microRNAs, and other proteins like enzymes. Through secretion of these signals, CAFs can establish the physical barrier surrounding cancer cells and thus directly supporting cancer progression via immune cell polarization, leading to a protumoral, immunosuppressive status (21, 31–33). Depending on the stage of tumor progression, CAFs contribute to the characteristics of the TME including the ECM through direct humoral interaction with TAMs (34). They remodel the ECM via qualitative and quantitative changes in the production of collagen, laminins, or fibronectins or tenascins through reorganization of protein synthesis and structure (12, 21). CAFs and cancer cells cooperate with each other through secretion of proteolytic enzymes such as matrix metalloproteinases (MMPs) that destroy the ECM and control the modification and cross-talk linking of ECM proteins (e.g., lysyl oxidases), leading to increased stiffness of ECM and its altered composition (21, 30, 35–38). This induces desmoplasia and fibrosis, establishing a physical barrier between tumor cells and therapeutic drugs as well as immune cells and enabling cancer cells to invade and metastasize (39).

CAFs may increase monocyte recruitment through secretion of monocyte chemoattractant protein-1 and stromal cell-derived factor 1 (SDF-1) and differentiation into TAMs, particularly M2 cells (35, 38). CAFs can promote tumor development by maintaining monocyte chemotactic protein-1-mediated macrophage infiltration and chronic inflammation and have been associated with infiltration of CD204^+^ TAMs (40, 41). CAFs and M2 macrophages were demonstrated to cooperate with each other during cancer progression, and they are able to alter each other’s functions through constant cross-talk (37, 42–45).

Finally, the TME restricts the entry of any cytotoxic antitumor substance or antitumor immune cells to the tumor cells by establishing cellular and noncellular barriers around the malignant cells (29, 36). Together with the vascular network, the ECM, and necrotic tissues, CAFs may shield tumor cells from outside signaling, completing the TME framework. **Table 1** lists different components of antitumorigenic and protumorigenic microenvironments. A remodeled TME with rewired macrophage function is considered one of the key mechanisms of resistance to chemotherapy and immune checkpoint inhibitors, which we characterize and discuss below.

## 2 Types of macrophages and their characteristics and impact on tumorigenesis

Macrophages and other myeloid cells constitute more than 50% of a tumor mass and are crucial to its development (31, 46). The significant infiltration of macrophages in tumor metastases has been recognized as an independent biomarker of poor prognosis (3, 11, 13, 14, 47–50). Although macrophages exhibit high heterogeneity, three main populations of macrophages can be distinguished: TAMs, tissue-resident macrophages, and MDSCs (51). Among these populations, TAMs are the most abundant infiltrating cells in the TME (52). Because of their extreme plasticity and ability to adapt to external stimuli, macrophages can differentiate into specific subpopulations in response to environmental changes, in a process known as polarization, and perform functions dictated by the environment (51–53).

The two main types of macrophages commonly recognized are M1, also referred to as classically activated macrophages, and M2, alternatively activated macrophages (53). Despite the considerable plasticity of macrophages and their capacity to differentiate through polarization, researchers have proposed using various markers to characterize and distinguish between M1 and M2 morphology (54, 55). The utilization of these markers has demonstrated that M1 macrophages are often characterized by the presence of CD68 and CD80 and exhibit high expression of the MHC-II complex (56), whereas M2 macrophages are characterized by high expression of CD23 [the low-affinity receptor for immunoglobulin (Ig)E], CD163 (hemoglobin scavenger receptor), CD204 (class A macrophage scavenger receptor, SR), or CD206 (mannose receptor, C type 1, MR); a low expression of the MHC-II complex; and expression of arginase 1 (21, 35, 36, 57).

In terms of their function, M1 macrophages are involved in immune defense against external pathogens and promoting antitumor immunity (2, 53). They exert their immunostimulatory and tumoricidal effects through the release of various chemicals and molecules, including lipopolysaccharides, IFN-γ, tumor necrosis factor (TNF)-α, IL-12, IL-18, reactive nitrogen and oxygen species, inducible nitric oxide synthase, CXCL9, CXCL10, and major histocompatibility complex (MHC)-II. Additionally, they participate in the process of antigen presentation (20, 58).

**Figure 1:**
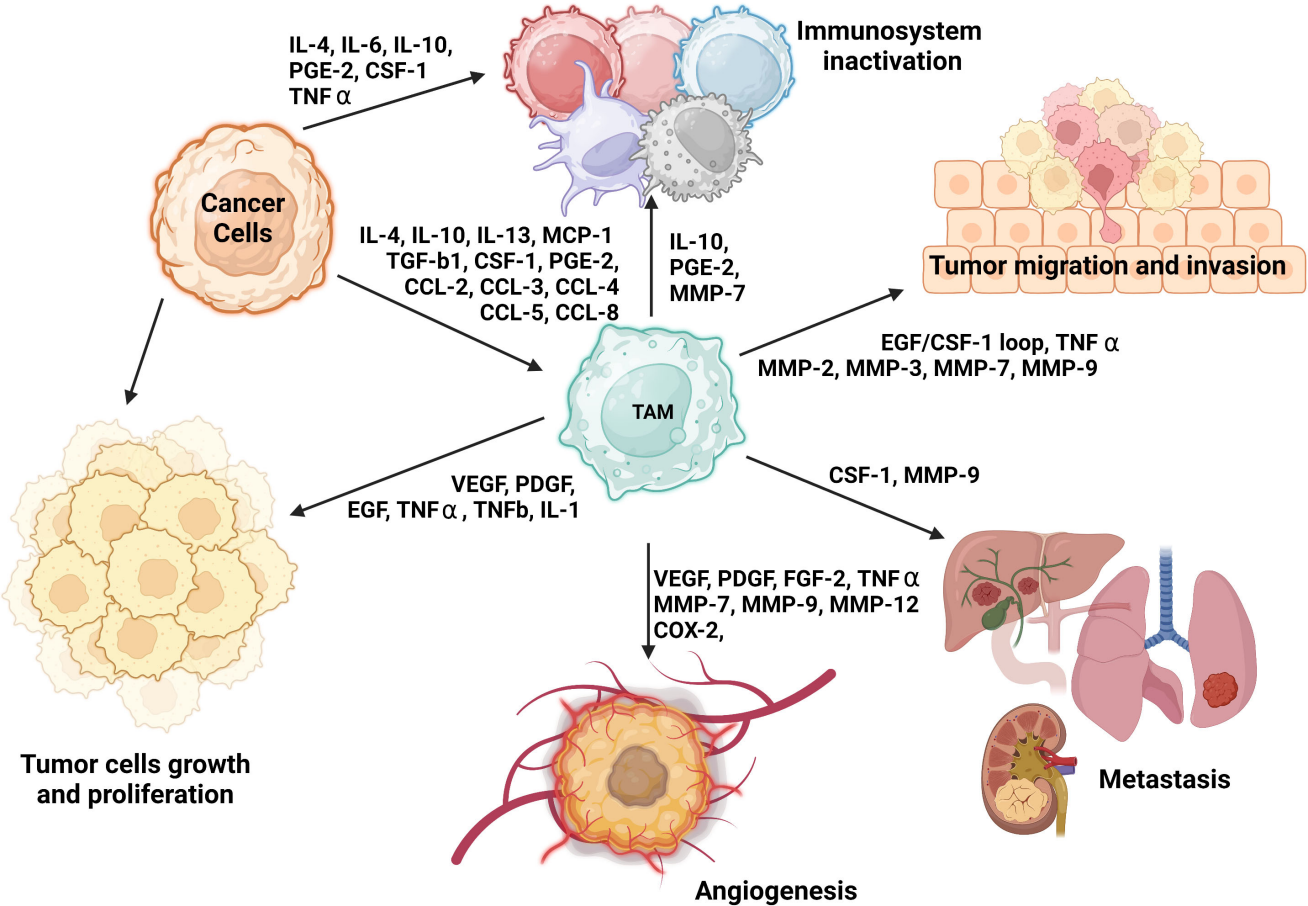
The role of M2 TAMs and their impact on tumorigenesis and immune system evasion. TAMs engage in several phases of tumorigenesis by secreting growth factors, chemokines, cytokines, and TGF-β (51, 60–63). These cells can foster a susceptible to modulation microenvironment by polarizing CD25^+^ T cells to Th2 and Treg phenotypes. They can also restrict the antitumor ability of NK cells and cytotoxic T cells (CD8^+^ T cells) by generating TGF-β (64). Additionally, TAMs may promote the invasion of cancer cells by producing EGF and CCL2 in the TME (65).

On the other hand, M2 macrophages, which naturally occur in normal physiological conditions, are involved in Th2-mediated immune response, particularly in humoral immunity, wound healing, and tissue remodeling (52). However, in the presence of tumor cells, alternatively activated M2 macrophages assume an immunosuppressive and tumor-promoting role (52). The characteristics of tumor-associated M2 macrophages are orchestrated by the action of IL-4, IL-10, IL-13, macrophage colony-stimulating factor 1 (CSF-1), CCL2, or VEGF-A (2, 22, 51, 53, 59) (**Figure 1**).

The specific polarization state of TAMs can be influenced by certain chemokines and other substances secreted by tumors. The expression pattern of surface markers in M2 macrophages is heavily influenced by the presence of IL-4, -10, and -13 or MMPs such as MMP-1, MMP-3, MMP-10, and MMP-14, which are secreted by the tumor. The levels of these factors can vary among organs and types of tumors (2, 51–53) (**Figure 1**). Further distinctions between M1 and M2 macrophages can be made based on the quality and quantity of secreted cytokines and chemokines. Upon exposure to inflammatory signals, M1 macrophages secrete IL-1β, IL-6, IL-12, IL-23, CXCL9, CXCL10, TNF-α, nitric oxide, and reactive oxygen species (52, 53, 55). In contrast, in response to secretion of cytokines by tumor cells, M2 macrophages may release hepatocyte growth factor, TGF-β, VEGF-A, FGF-2, platelet-derived growth factors, placental growth factor, insulin-like growth factor-1, IL-1, IL-10, IL-8, CCL17, CCL22, SDF-1 (CXCL12), PD-L1, PD-L2, arginase, and prostaglandin E2 (22, 48, 59, 66–68). Additionally, M2 macrophages can synthesize and release MMP-2, MMP-7, MMP-9, MMP-13, cathepsin B and S, and serine proteolytic enzymes that break down the ECM as well as secrete growth factors necessary for EC proliferation and microvessel development (48), as shown in **Figure 1**.

Notably, researchers have shown M1 and M2 macrophages to have distinct angiogenic potential *in vitro*, with the M2 phenotype expressing more proangiogenic cytokines and other growth factors than does the M1 phenotype, which is discussed below in detail (48). Furthermore, M1 and M2 macrophages can be distinguished by their metabolic state. M2 macrophages mainly have a preponderance of glycolysis, fatty acid synthesis, and the pentose phosphate pathway, whereas M2 macrophages largely depend on oxidative phosphorylation (OXPHOS) for their biosynthetic and bioenergetic needs (69). TAMs are closely involved in angiogenesis, suppression of the immune system, impairment of the other immune cells’ function, and support of tumor-cell metastasis. TAMs consist mostly of M2 macrophages and are thus thought to resemble M2 macrophages with their wide array of secreted cytokines, chemokines, and enzymes, and Th2 immune response (22, 70); therefore for the purpose of simplicity, we will further refer to TAMs or tumor-associated M2 macrophages equally. **Figure 1** provides an overview of the various roles of M2 TAMs in tumorigenesis, including an immune system inactivation, which is discussed in detail in the next section.

## 3 The role of TAMs in suppression of immune responses

**Figure 2:**
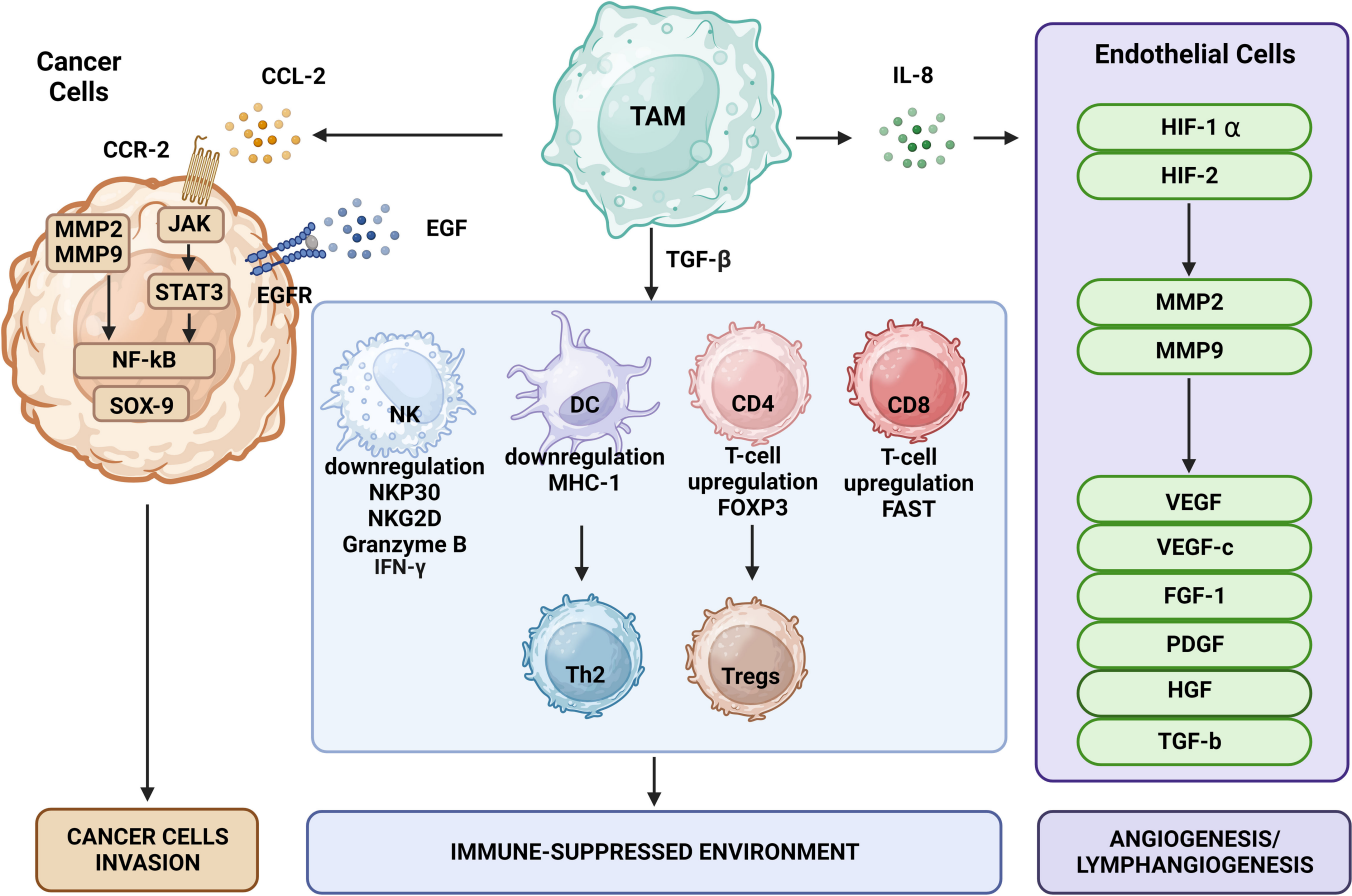
The effects of TAMs on tumor cells include promotion of tumor growth, angiogenesis, induction of tumor infiltration and immune suppression by Tregs, metabolic deprivation of T cells, inactivation of T cells, induction of growth and proliferation of cancer stem cells, EMT, invasion, migration, and metastasis. TAMs encourage the growth of tumors by secreting certain substances and expressing specific proteins. MMPs, CSF-1, and EGF produced by TAMs promote tumor invasion and migration. Moreover, TAMs release VEGF and platelet-derived growth factor, which encourage angiogenesis and tumor growth.

Immune surveillance against cancer involves immune cells such as CD4^+^ Th cells, CD8^+^ cytotoxic T cells, NK cells, and DCs (**Figure 2**). TAMs disrupt the function of these cells via secretion of specific cytokines. TGF-β is one of the key regulators of immunosuppression that may prevent the production of cytotoxicity-promoting receptors like natural cytotoxicity triggering receptor 3 (also known as NKp30) and NK group 2 member D protein upon binding of its receptors on the surface of NK cells (71). TGF-β may also affect T cells by impairing their ability to express lysing genes like granzyme A and B together with IFN-γ and FAS ligand, thus inhibiting their cytotoxic function. TGF-β also may induce expression of FOXP3 in CD4^+^CD25^+^ T cells, contributing to recruitment and an increase in the pool of Tregs in the TME (72), which can weaken the immune functions of CD4^+^ and CD8^+^ T cells (73). Thymus-derived CD4^+^CD25^+^FOXP3^+^ Tregs may increase the pool of CD206^+^CD163^+^ macrophages that differentiate from monocytes and upregulate CCL18 and IL-1Ra produced by macrophages (74).

By triggering CD4^+^ T cells to differentiate into the Th2 phenotype, TGF-β and its receptor in DCs decrease adaptive immune responses through apoptosis induction and reduction of antigen-presentation ability. Thus, TGF-β changes the balance between Th1 and Th2 cells in favor of Th2 cells and enhances the immunosuppressive structure of the TME (63, 75, 76). In addition to interacting with local immune cells in an inflammatory TME, secreted TGF-β may stimulate tumor cells and MDSCs to release IL-10. The latter could be further enhanced by synergistic interaction of IL-10 with TGF-β and prostaglandin E2 via EP2 and EP4 receptors, which direct TAMs to further sustain the secretion of IL-10 (77). This cascade continues to transform naïve T cells into Tregs and inhibit the antitumor immunity maintained by NK cells (78).

IL-10 may decrease the production of proinflammatory cytokines such as IL-6, TNF-α, and IFN-γ and thus promote polarization of macrophages toward the protumorigenic M2 phenotype and thus ultimately enable tumor cells to evade immune surveillance (79). IL-10 may also inhibit or downregulate macrophage IL-2 production and thus induce macrophage polarization into the M2 phenotype (79). Furthermore, secreted IL-10 may induce release of PD-L1 and cytotoxic T-lymphocyte-associated antigen-4 as well as expression and activation of the corresponding receptors to further reduce the antitumor activity of T cells. The binding of PD-L1, followed by its activation of programmed cell death protein 1 (PD-1; CD279), or receptors B7-1 (CD80), and B7-2 (CD86) on the surface of TAMs, DCs, and B cells, triggers inhibitory signals, leading to a state of immunological tolerance and negative regulation of T-cell immune response, including apoptosis, anergy, and exhaustion (80–82). PD-L1’s activation of CD80/CD86 and CD28 receptors also causes decreased proliferation, cytokine production, and T-cell anergy (80–82). Thus, to reactivate the immune response and enhance antitumor results of anti-PD1 therapy, blocking or reversing these interactions among T cells and macrophages is crucial. This immunosuppression mechanism plays a crucial role in tumor immune evasion.

TAMs also subvert immune surveillance by expressing cell surface proteins or releasing other soluble factors such as arginase 1, indoleamine 2,3-dioxygnease, and inducible nitric oxide synthase, which are oxygen and nitrogen radicals that harbor immunosuppressive functions and inhibit proliferation of NK and T cells (83, 84). TAMs restrained T-cell-specific response and crippled CD8^+^ T-cell proliferation and killing activity via the release of extracellular vesicles (EVs), which led to tumor immune evasion (85, 86). Investigators showed that T-cell exhaustion was induced by leukemia-cell-derived EVs transporting the microRNA miR-21-5p. EVs harboring miR-21-5p also enhanced CD8^+^ T-cell exhaustion in mice with primary hepatocellular carcinoma by targeting of YOD1 and activating the YAP/β-catenin signaling pathway (87).

To induce macrophage polarization toward the M2 phenotype, renal cell carcinoma (RCC)-derived EVs containing *lncARSR* delivered to macrophages acted as competing endogenous RNA for the microRNAs *miR-34*/*miR-449*, thus increasing signal transducer and activator of transcription 3 (STAT3) expression as the primary type of signaling of macrophage polarization (88). In addition, glioblastoma-derived EVs reprogram M1 macrophages to become TAMs and enhance protumor functions of the M2 macrophages (89). Similarly, M2-polarized TAM-derived EVs showed an activity to influence proliferation, migration, invasion, and tumorigenesis of meningioma tumors through activation of TGF-β signaling, and with delivery of *oncomiR-21* and *AKT*, *STAT3*, *MTOR*, and *ACTB* mRNA expression showed to support progression, migration, tumor sphere generation, and cisplatin resistance of bladder cancer (52, 90). Furthermore, TAM-derived exosomes promote the migration, growth, and proliferation of glioblastoma cells (50). Finally, EC-derived EVs in the TME were shown to recruit macrophages to tumors, resulting in transferring microRNAs via EVs to M2-like macrophages and causing an immunomodulatory phenotype that permits tumor growth (91).

In summary, TAMs govern immunosuppression by inducing phenotypic changes in other immune cells, recruitment and migration of myeloid DCs, stimulation of immunosuppressive cells, and production of chemokines and cytokines that regulate both the function of immunosuppressive cells and promotion of tumor-cell growth, thus impairing the effectiveness of chemotherapy and contributing to chemotherapy and immunotherapy resistance. Hence, targeting TAMs may enhance chemotherapy and immune therapy responses of tumor cells by boosting the immune system.

## 4 The roles of TAMs in tumor cell initiation, growth, and progression

Tumorigenesis is strongly associated with inflammation. In the process of establishing an inflammatory environment, TAMs play an essential role (53, 73) by producing mediators that remodel the TME or directly support tumor cell proliferation, protect tumor cells from apoptosis, and modulate tissue composition to favor cell migration, invasion, and metastasis. Investigators demonstrated these functions of TAMs in solid tumors such as colon and gastric cancer (73, 92, 93), in which underlying chronic inflammation or activation of specific oncogenes may cause activation and expression of proinflammatory transcription factors. The most examined transcription factors associated with inflammation include nuclear factor (NF)-κB, STAT3, hypoxia-inducible factor (HIF)-1α, and HIF-2 (73). Activation of these signaling pathways in cancer cells leads to a cascade of events with the release of cytokines and chemokines such as TNF-α and IL-6, which authors reported led to the recruitment, migration, and polarization of MDSCs and monocytes; differentiation of monocytes to macrophages; and ultimately the polarization of macrophages toward the M2 phenotype (56, 73, 79).

Macrophages might initially produce several proinflammatory mediators (IL-6, TNF-α, and IFN-γ), growth factors (EGF and Wnt), enzymes like proteases, and free radicals. This cocktail of substances, chemokines, and growth factors boosts the creation of a mutagenic microenvironment that favors and facilitates cancer initiation, and in consequence macrophage polarization toward M2 phenotype (81, 94). TAMs may also release other ILs such as IL-6, IL-17, and IL-23 that can support tumor growth and progression as shown in models of colon cancer and hepatocellular carcinoma, in which tumor progression was associated with activation of the STAT3 signaling pathway (95, 96). In summary, as depicted in **Figures 1**, **2**, the impact of TAMs on the initiation of tumorigenesis involves secretion of diverse factors and chemokines that lead to an accelerated tumor expansion and spread, which is discussed in the next section.

## 5 The role of TAMs in angiogenesis and lymphangiogenesis

**Figure 3:**
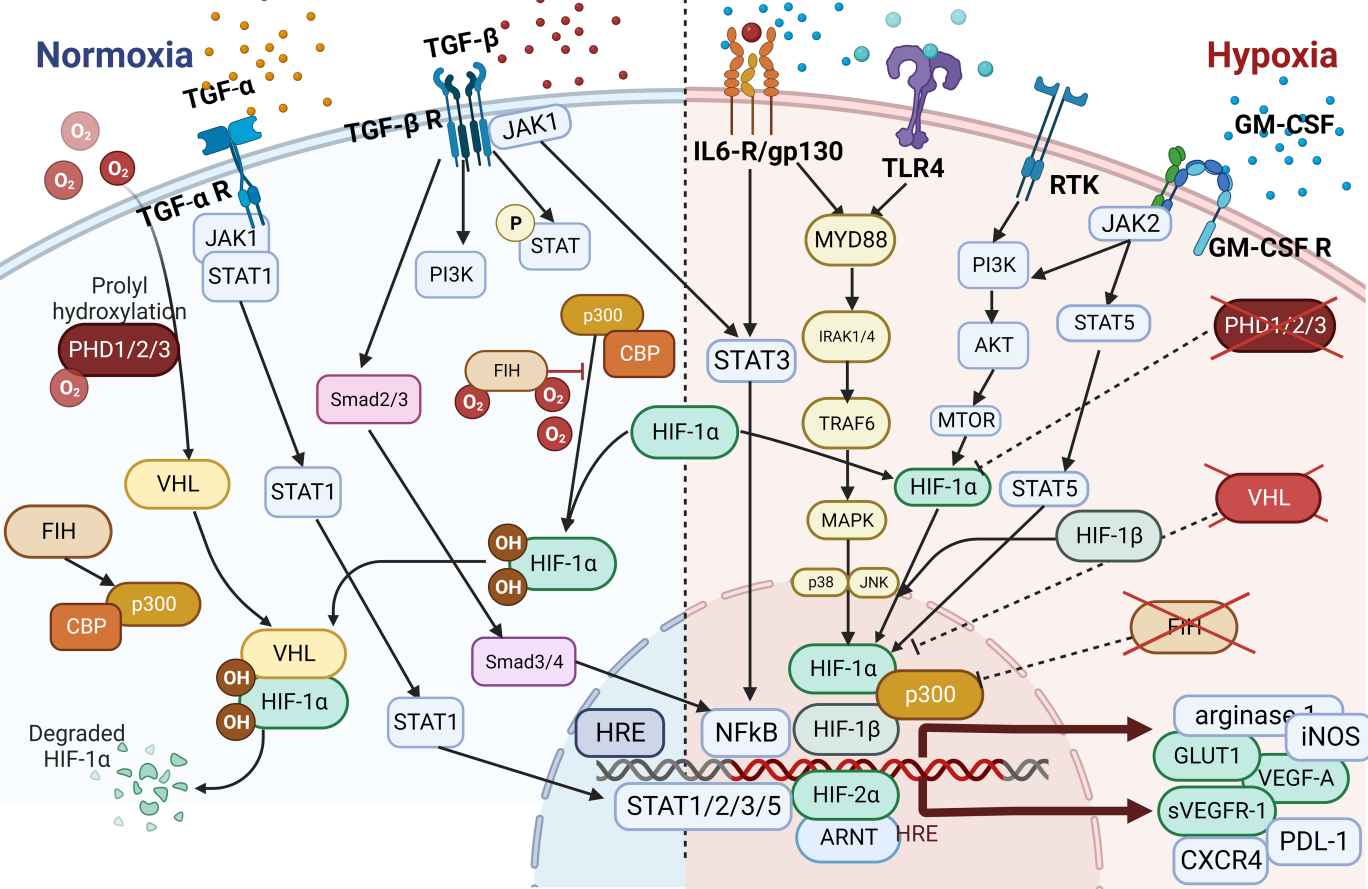
Effects of hypoxia and acidosis on TAMs. The TME is influenced by hypoxia and acidosis, which significantly impact TAMs. Hypoxia induces an M2-like phenotype, supporting tumor growth, whereas acidosis shifts TAMs toward immunosuppression and reduces proinflammatory cytokine output. These factors contribute to tissue remodeling, ECM disintegration, and angiogenesis (92, 117–119). Together, hypoxia and acidosis shape TAM activities, promoting tumor growth, blood vessel formation, and immune system evasion (119).

In addition to tumor initiation and growth-supporting activities, TAMs can promote neovascularization to maintain the supply of nutrients and growth factors necessary for increasing the energy and biosynthesis demands of tumor cells required for expansion, invasion, and metastasis. In this context, angiogenesis and lymphangiogenesis are often discussed in association with factors like hypoxia, acidosis, and hyperosmotic pressure that, together with angiogenic factors such as VEGF-A (97–100), TGF-β (63), cyclooxygenase-2, placental growth factor, FGF-2 (62), EGF, platelet-derived growth factor, insulin-like growth factor-1, angiotensin-1, and chemokines like SDF-1, stimulate these processes (**Figure 1**) (48, 66, 68, 92, 101–104). The precise mechanism underlying cell-to-cell contacts between ECs and macrophage subsets as well as that underlying macrophage-stimulated angiogenesis has yet to be fully determined. However, TAMs may contribute to these processes by controlling responses to inflammatory stimuli through the release of angiogenesis- and lymphangiogenesis-stimulating factors such as VEGF-C and VEGF-D (62, 105–108). VEGF-C-mediated lymphangiogenesis may also result from a process associated with overexpression of MMP-2, MMP-3, and MMP-9 or MMP-13 that degrades the ECM and thereby indirectly facilitates angiogenic invasion, linking neovascularization with TME and matrix remodeling (48, 62, 68, 104, 109, 110). Production of proangiogenic factors such as VEGF and FGF-2 is commonly increased in hypoxic areas and has been linked to elevated expression of HIF-1α, a transcription factor that plays a central role in regulating the activation of genes in response to decreased/low oxygen levels in cells (91, 111, 112). Under elevated hypoxic conditions, due to the uncontrolled cell growth and tumor expansion especially in the middle of the tumor mass, HIF-1α was shown to interact with the transcriptional co-factor p300/CBP, activating a wide range of genes, upregulating expression of the SLC2A1/GLUT1 receptor, and increasing glycolytic activity (46, 111–114). This in consequence leads to increasing distance between blood vessels and individual cells within the tumor mass, reducing an intratumoral oxygen level, and thus deepening further the level of hypoxia within the tumor due to limitations in oxygen diffusion and oxygen availability for selected cells (115). Increased hypoxia together with elevated glycolytic activity as shown for most of solid tumors, increased the production and secretion of VEGFs, thereby promoting neovascularization and finally increasing the release of TGF-α/β to induce angiogenesis and impediment of immune cells’ tumor growth–inhibitory properties (59, 116) as shown in **Figures 1**, **3** (92, 119, 120).

Researchers also demonstrated upregulated expression of VEGF-A in tyrosine kinase with immunoglobulin and EGF homology domain 2 (Tie2)-positive macrophages. VEGF-A secreted by Tie-2-expressing macrophages (TEMs) induced proliferation of ECs, which led to tumor angiogenesis (121). Furthermore, Tie2 on TEMs binds to angiopoietins 1–4, which initiates vascular development or neoangiogenesis (122, 123) and is a homing mechanism for ECs and vessel development (122, 123). Of note, Tie2 is frequently co-expressed with CXCR4, a chemokine receptor for SDF-1 linked to cell migration (124, 125). SDF-1 is a membrane-bound or released chemoattractant cytokine that promotes inflammation, thereby primarily attracting leukocytes, hematopoietic stem cells from adult bone marrow, and macrophages (126). SDF-1 is predominantly expressed by ECs (127), and its expression and secretion results in consistent recruitment of CD11b^+^ monocytes/macrophages and retention of these cells in the tumor environment (128). Besides the presence of Tie2 (109), CXCR4 or CD11b (CD18/MAC-1) TAMs express and secrete angiogenic cytokines like MMP-9 and MMP-13 (50) stimulating further the process of neovascularization (129). Of note, during brain vascularization, yolk-sac-derived macrophages expressing Tie2 make up most of tissue macrophages and work with the endothelial tip cells to enhance vascular anastomosis following VEGF-mediated tip-cell proliferation and sprout formation (50). Also, EGF secretion by TAMs may activate EGFR on tumor cells, further upregulate VEGF/VEGFR signaling, and thus increase cancer cell proliferation and invasion (130). TAMs may also promote angiogenesis by increasing the secretion of TGF-β and IL-10, resulting in the proliferation of vessel ECs (131). Stimulation of ECs by Wnt family ligand 7B (WNT7B) aberrantly expressed in TAMs, which regulates the Wnt/β-catenin signaling pathway and VEGF production, and thereby triggers angiogenesis, tumor progression, growth, tumor cell invasion, and metastasis, was demonstrated in models of luminal breast cancer (73, 132, 133). Furthermore, myeloid Wnt7b caused an overexpression of VEGF-A in ECs, leading to angiogenic switching and tumor neovascularization (132).

In summary, the contribution of TAMs to tumor neovascularization provides solid evidence that TAM targeting may diminish or reduce tumor progression and metastasis directly by reducing TAM abundance and indirectly by impairing the release of angiogenesis-stimulating factors. Combinatorial approaches to targeting tumor cells such as classical chemotherapy together with strategies aimed at targeting TAMs and neoangiogenesis may be superior to chemotherapy or immunotherapy alone. Alternatively, approaches targeting TAMs combined with immunotherapy targeting EGFR or VEGFR and/or HIF-1/2 may warrant preclinical and clinical testing and inhibit tumor expansion.

## 6 The role of TAMs in tumor metastasis and invasion

The migration of tumor cells to ectopic sites requires both angiogenesis and lymphangiogenesis (134, 135). In line with TAMs’ involvement in angiogenesis, a plethora of evidence has emphasized the importance of TAMs to tumor invasion and metastasis (136). For example, neovascularization is essential for metastasis, enabling cancer cells to spread from the primary tumor to distant sites. It enables cancer cells to enter blood or lymphatic vessels, allowing them to adhere to vessel’s walls, penetrate barriers, and establish secondary tumors. The tumor vasculature’s permeability and angiogenesis create a supportive microenvironment for cancer cell survival and growth. Given that metastasis is the main cause of death in cancer patients, targeting tumors at this stage is an urgent need. A common feature of cancer cells is their ability to move and release digestive enzymes that enable escape from the primary tumor and to break into the vascular and lymphoid systems to further colonize distant sites (85, 93, 133, 137).

Invasion and metastasis can also be conferred via initiation of epithelial-to-mesenchymal transition (EMT), a process enabling epithelial cells to acquire mesenchymal features (138). EMT is a crucial biological process in cancer development in which epithelial cells become more motile and invasive mesenchymal-like cells. This process facilitates invasion, metastasis, and therapeutic resistance of cancer cells. Cancer cells thus lose adhesion, become more motile, and resist apoptosis. EMT also aids in angiogenesis and immune evasion, making tumors more resistant to various treatments. EMT is linked to resistance to various treatments, including chemotherapies and targeted therapies. Understanding and targeting EMT in cancer research may lead to potential techniques for reducing metastasis, increasing therapy responses, and improving outcomes. Recent studies demonstrated that EMT is regulated by TAMs, further facilitating metastasis (132, 139).

TAMs interact with cancer cells, promoting EMT-related genetic alterations and facilitating cell migration and invasion. They also contribute to ECM remodeling and promote an immunosuppressive milieu, supporting EMT indirectly by suppressing immune responses. This interaction creates an EMT-friendly microenvironment, enabling cancer cells to penetrate tissues, enter the circulation, and metastasize to other organs. EGF production by tumor-infiltrating M2 TAMs within the TME can stimulate the NF-κB, STAT3, EGFR, and extracellular signal-regulated kinase signaling axes in tumor cells, promoting their invasive traits (140, 141). For instance, TAMs increase cancer cell invasion and capability for metastasis through induction of EMT by interfering with JAK2/STAT3/miR-506-3p/FoxQ1 regulation of colorectal cancer development (139). Additionally, EGF may prevent expression of the long noncoding RNA LIMIT, increasing the capacity for cancer cells to move (142).

The expression of EGF by TAMs may be adversely affected by CSF-1 synthesized by tumor cells, which may enhance the metastatic potential of tumor cells (143). EGF secreted by TAMs activates the EGFR/extracellular signal-regulated kinase 1/2 signal pathway in some types of cancer cells, which results in the promotion of EMT (144). Additionally, authors suggested that TGF-β generated by these TAMs in lung cancers boosts the expression of SOX9 and triggers EMT, thereby causing tumor cell migration (145). TAMs also support tumor metastasis through increased expression and release of MMPs such as MMP-2 and MMP-9 (143). MMPs together with VEGF-C, activates the CCL2/CCR2 signaling pathway and attracts circulating monocytes into the TME, thereby promoting tumor growth, expansion, and metastasis (132, 137, 146). These infiltrating monocytes may facilitate tumor growth, expansion, and metastasis by releasing tumor-promoting factors. For example, monocytes can secrete growth factors such as VEGF-C, which induce angiogenesis and lymphangiogenesis, resulting in the creation of new blood vessels that deliver oxygen and nutrients to tumors. These cells can also produce cytokines and chemokines, which attract additional immune cells to the TME, where they dampen the immune response and promote tumor growth. Furthermore, monocytes can develop into TAMs, which are already demonstrated to enhance tumor progression by releasing a variety of substances that encourage tumor cell proliferation, invasion, and metastasis (147).

Activation of the JAK2/STAT3/miR-506-3p/FoxQ1 axis may also result in the generation of CCL2 and thereby facilitate the recruitment of macrophages (139). Furthermore, increased CCL2 expression in the TME is accompanied by increased CCR2 expression on TAMs and by the polarization of macrophages toward the M2 phenotype, whereas CCL2 overexpression and high TCF4 expression correlate with cancer metastasis to lymph nodes and have been linked to poor prognosis because the TCF4/CCL2/CCR2 regulation axis regulated TAM polarization (146). Of note, preclinical studies demonstrated M2 macrophages’ potent induction of an invasive phenotype in previously healthy epithelial cells through the release of CCL2 and upregulation of endoplasmic reticulum oxidoreductase 1α as well as MMP-9, leading to acquisition of an invasive EMT phenotype (101, 148–151). TAMs may also release CCL5, which, through activation of the β-catenin/STAT3 signaling pathway, significantly promoted invasion, metastasis, and EMT in studies using prostate cancer cells (24, 86, 146, 150–152). Of note, CCL5, which is released by malignant phyllodes tumors, can trigger recruitment and repolarization of TAMs through activation of the CCR5 receptor and the AKT signaling pathway.

Furthermore, TAM-secreted CCL18 can bind to the membrane-associated phosphatidylinositol transfer protein 3 receptor, which further facilitates differentiation and invasion of myofibroblasts (83). Infiltration of TAMs and invasion and metastasis of colorectal cancer cells were promoted by the phosphatase of regenerating liver-3 (PRL3)-stimulated upregulation of cytokine CCL26 and activation of CCR3 receptor (85). Whereas EMT and metastasis induction in a model of non-small cell lung cancer (NSCLC) were facilitated by upregulation of αβ-crystallin upon co-culture of TAMs with cancer cells (153), phosphorylated STAT3 with upregulation of cyclooxygenase-2 and MMP-9 led to EMT induction, invasion, and metastasis in animal models of osteosarcoma (154).

Taken together, these findings demonstrate that TAMs can express and release a variety of factors to induce EMT. Therefore, targeting TAMs, even in advanced stages of cancer development, may have life-extending benefits for patients.

## 7 The role of TAMs in chemoresistance

**Figure 4:**
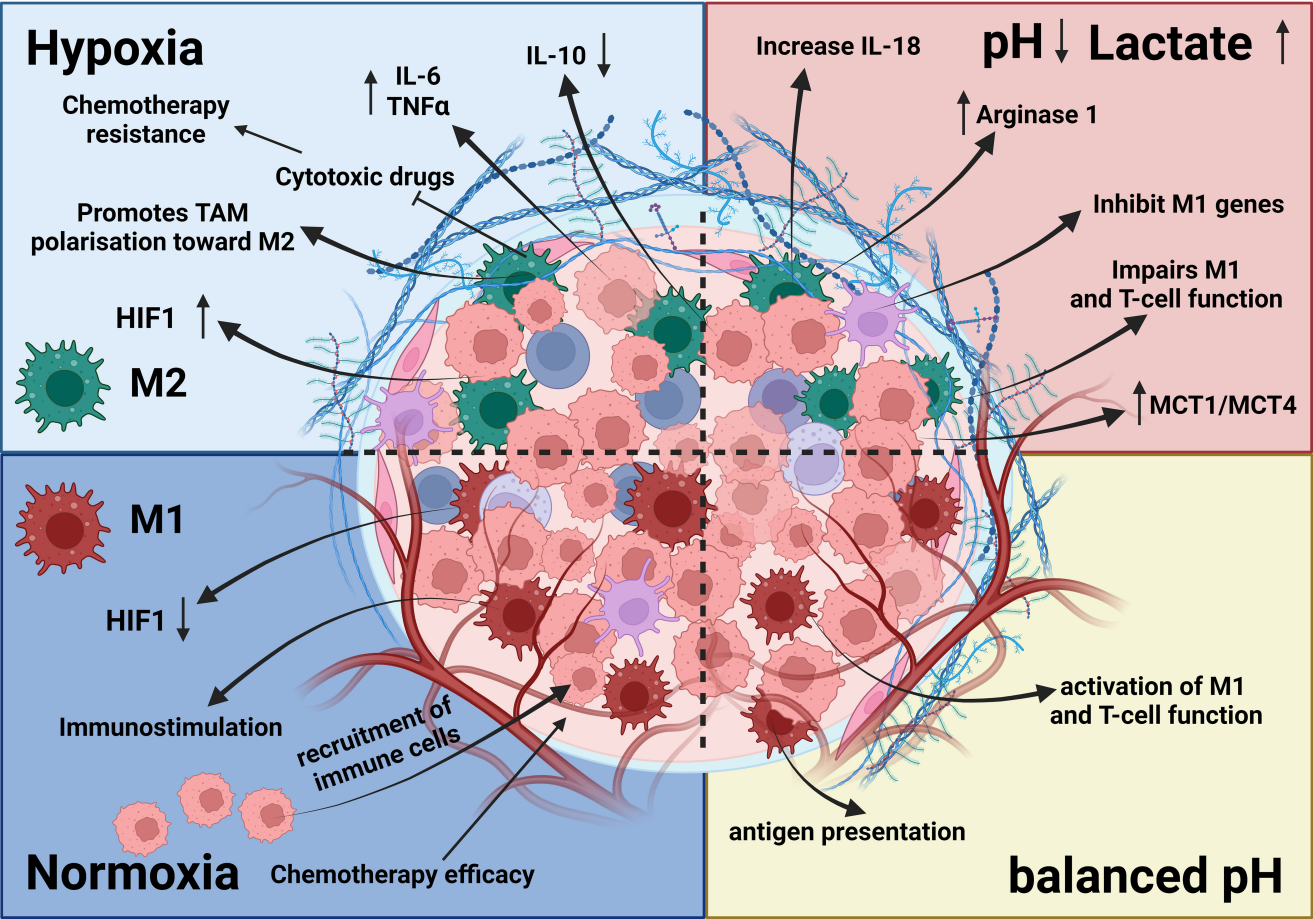
The Hypoxia pathway. Overactivated STAT3 and NF-κB activate the transcription of HIF-1α, which has resulted in the overexpression of HIF-1α (116). In combination with HIF-1β, HIF-1α triggers the transcription of TGF-α and TGF-β. Moreover, HIF-1α indirectly activates VEGF, leading to angiogenesis via overactivation of TGF-α. HIF is a transcription factor that plays a central role in regulating the activation of genes in response to low oxygen levels in cells. HIF-dependent mechanisms influence gene expression by affecting epigenetic factors such as DNA methylation and histone acetylation (155). HIF binds to DNA and associates with distinct nuclear co-factors under low-oxygen conditions. Oxygen depletion causes HIF-α to interact with the transcriptional co-factor p300/CBP. This association activates a wide range of genes, initiating diverse adaptive processes such as glycolysis (SLC2A1/GULT1), angiogenesis (VEGF-A), and angiogenesis and loss of growth-inhibitory effects (TGFα/β) (156, 157).

Depending on the tumor type, most cancer treatments consist of a combination of chemotherapy, immune therapy, hormonal therapy, immune checkpoint blockade (ICB), and/or radiotherapy. Acquired resistance to treatment is the most common reason for treatment failure, and researchers have extensively investigated the contribution of TMEs including TAMs to treatment resistance. Macrophages can be prompted by their environment to adopt multiple phenotypes, and most TAMs are commonly polarized toward a cancer-promoting phenotype, which confers treatment resistance (102, 152). Treatment resistance may either reduce or completely impair the effectiveness of therapy. Investigators have identified several mechanisms of resistance conferred by TAMs. Changes in the profiles of secreted cytokines, expression of different receptors, activation of transcription factors and signaling pathways mostly associated with inflammation or hypoxia, changes in polarization of TAMs, rewiring of metabolism, and initiation of dynamic changes in the microvasculature are only some of the resistance mechanisms (**Figure 4**). Overall, TAMs limit the effectiveness of cancer therapies, triggering detrimental reactive responses to tumor-induced tissue damage cues and rapidly reprogramming the TME toward a proremodeling state (53, 56, 120, 158). For instance, in prostate cancer models, secretion of CCL5, activation of STAT3, and upregulation of the transcription factor Nanog resulted in chemotherapeutic drug resistance, whereas secretion of CXCL12 and activation of CXCR4 by TAMs occurred following combined docetaxel/androgen deprivation therapy in cases of castration-resistant prostate cancer tumors with poor response (84, 103, 159).

Researchers found markedly greater TAM abundance in patients with NSCLC who experienced progressive disease upon treatment with an EGFR tyrosine kinase inhibitor (137) than in those with nonprogressive disease. Moreover, as described previously, high TAM counts were significantly associated with poor progression-free and overall survival, suggesting that TAMs are related to reduced treatment responsiveness after administration of not only EGFR tyrosine kinase inhibitors but also several commonly used treatment combinations (159) and mediate resistance to antiangiogenic therapies via compensatory pathways such as cathepsin B and angiopoietin-2. Also, TAMs are key players in the antitumor activity of selected monoclonal antibodies (mAbs) such as rituximab (anti-CD20), trastuzumab (anti-HER2), cetuximab (anti-EGFR), and daratumumab (anti-CD38), as they express FcγR to perform tumor-cell killing and phagocytosis (127, 160). However, functional polymorphisms in human FcγRIIIA that affect the killing ability of macrophages correlate with low rates of response to treatment with mAbs in patients with lymphoma, breast cancer, or myeloma (127, 160).

In addition, the effects of hormonal therapy on disease progression and survival are impacted by inflammatory pathways orchestrated by macrophages. Inflammatory cytokines such as IL-1 and IL-6 can activate estrogen or androgen receptor signaling on tumor cells, linking inflammation to tumor growth and endocrine resistance (159). A new level of therapeutic intervention was introduced with the development of ICB. However, shortly after its introduction into the therapeutic armament, authors reported new resistance mechanisms mainly driven by macrophages. For instance, as key cell types participating in tumor-extrinsic pathways of primary and adaptive resistance, macrophages express several immunosuppressive molecules, including checkpoint ligands such as PD-L1, PD-L2, poliovirus receptor (CD155), and TIGIT ligands. Researchers showed these and other molecules to be overexpressed and to impede the efficacy of ICB for NSCLC and other types of cancer (83, 84, 151). Also, whereas PD-L1 expression in tumor-infiltrating immune cells but not macrophages correlated with positive response to anti-PD-L1/2 therapy, expression of PD-1 in macrophages was negatively correlated with their ability to phagocytose tumor cells (58, 81, 161, 162). Another inhibitory receptor found on macrophages is VISTA, which cooperates with negative regulators of T and NK cells such as P-selectin glycoprotein ligand 1 and acts as a T-cell checkpoint-inhibitory ligand. Thus, targeting VISTA with mAbs led to transcriptional and functional changes that produced increased antigen presentation, activation, and migration (22, 163). Another aspect of resistance to ICB is the cellular composition of tumors. The presence of tumor-infiltrating neutrophils together with tumor-infiltrating macrophages accompanied by T-cell elimination/depletion has contributed to the lack of response of liver cancer cells to ICB (164). For instance, abundant M2 macrophages in renal cell cancer were associated with resistance to ICB. In particular, the presence of a macrophage subpopulation expressing TIM4 suppressed CD8^+^ T-cell responses, impairing the efficacy of ICB. However, ICB efficacy may be restored by targeting TIM4^+^ macrophages via anti-TIM4 antibody-mediated blockade (165).

Additionally, a new dimension of complexity in the effectiveness of and resistance to immunotherapies was revealed by studies of the microbiome, suggesting that the specific composition of the microbiome shapes the components of the TEM and thus enhances or impairs therapy response. The composition of the microbiota and the cellular composition of the TEM may result from complex cross-talk and exchange of cytokines and oncometabolites among the microbiota, tumor cells, and cellular immune environment. For instance, abundant and diverse gut bacteria enriched for Bacteroides species, shaped tumor myeloid infiltration, and thus increased the effectiveness of anti-cytotoxic T**-**lymphocyte-associated antigen and anti-PD-1 therapy for melanoma (166). Taken together, these findings suggest that macrophages, particularly TAMs, have an important influence on the activity of chemotherapy, radiotherapy, antiangiogenic agents, hormonal therapy, and ICB. Their role is complex, as they frequently serve as inhibitors of antineoplastic activity. Despite progress in dissecting the role of macrophages in conventional antineoplastic treatment modalities, the actual translation of these findings into more effective cancer treatments remains challenging. Depletion of macrophages can potentiate various chemotherapeutic and immunotherapeutic strategies. Several preclinical and clinical trials combining different therapeutic strategies, such as immune checkpoint inhibitors and anti-CSF-1R antibodies or other TAM-centered therapeutic strategies in combination with chemotherapy, are currently under way and are discussed below.

## 8 TAM-targeted therapies

**Figure 5:**
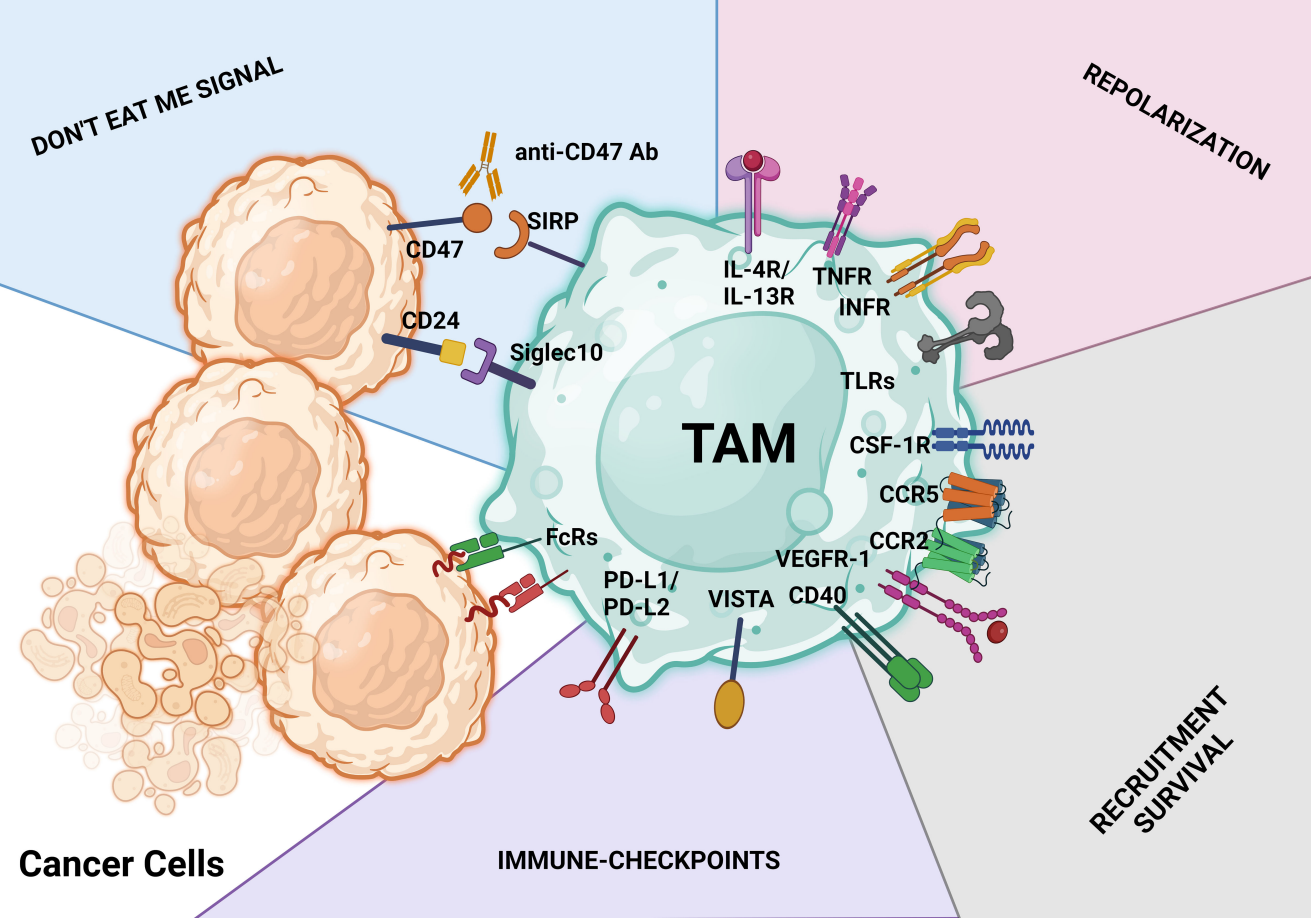
TAM-targeting strategies. These treatment approaches aim to either activate the antitumor behavior of TAMs or limit macrophage infiltration, survival, and protumoral actions. Identification of therapeutic antibodies using Fc receptors (FcRs) on TAMs is a key step in the macrophage-mediated antibody-dependent cellular cytotoxicity process. The CD47/SIRPα axis and CD24/SIGLEC10 pathway are both parts of the don’t eat me signal for tumor cells. Activating macrophage-mediated antibody-dependent cellular cytotoxicity phagocytosis is possible with antibodies against the CD47/SIRPα and CD24/SIGLEC10 pathways (antibody-dependent cellular phagocytosis). Don’t eat me signal pathways, repolarization, limiting and reducing the infiltration and survival of tumor cells, and ICB with antibodies are just a few of the major therapeutic approaches used to target TAMs.

Macrophages, the most prevalent immune cells within the TME, have a dual function in immunomodulation (19, 51). As discussed above, macrophages in cancer patients are an incredibly diverse mixture ranging from tumor suppressors (M1 phenotype) to tumor protectors (M2 phenotype) (19). Via sequestration of the release of proinflammatory cytokines and display of more than immunostimulatory markers, classically activated macrophages (M1 phenotype) support anticancer immunity (6, 19, 51). In contrast, M2 macrophages, which constitute most of TAMs, have a low antigen-presenting capacity and strong immunosuppressive features and produce higher numbers of proangiogenic cytokines than M1 macrophages (103, 167). Thus, limiting the number of TAMs or switching TAMs within the TME to the M1 phenotype is essential for cancer therapy because TAMs’ overall activity promotes tumor development and metastasis (19, 51). **Figure 5** summarizes selected therapeutic strategies targeting TAMs.

### 8.1 Blockade of TAMs migration/depletion of TAMs from the TME

#### 8.1.1 The CCL2/CCR2 axis

Blocking the CCL2 or CCR2 signaling pathway, an axis that draws circulating monocytes into the TME and induces their differentiation into macrophages, is one way to eliminate TAMs from the TME (101, 148–151). CCL2 blockade can stop tumor spread, angiogenesis, and growth, and researchers have demonstrated CCL2 restriction in animal studies to increase the antitumor effects of cytotoxic T lymphocytes and decrease the number of TAMs in the TME (168). Additionally, a CCR2 antagonist has exhibited tumor-burden-reducing efficacy in animal models of adenoid cystic carcinoma of the salivary glands by reducing the number of infiltrated TAMs (168). Studies demonstrated that targeting the CCL2/CCR2 axis with the antibody carlumab (CNTO 888) as well as with a specific inhibitor of the CCR2 receptor (PF-04136309) specifically blocks the CCL2-mediated activation and migration of macrophages into tumors and tumor’s infiltration by macrophages in patients with diverse types of cancers (149).

#### 8.1.2 CSF-1 and CSF-1R

Another valuable target for the removal of TAMs from the TME is CSF-1R. CSF-1 is a cytokine that is essential for the survival, proliferation, and differentiation of mononuclear phagocytes (84, 159, 169). CSF-1R is a tyrosine kinase transmembrane receptor that belongs to the CSF-1/platelet-derived growth factor receptor family of protein tyrosine kinases. It has an important role in the formation and maintenance of microglia in the brain (84, 159, 169, 170). CSF-1R promotes myeloid cell survival when activated by two ligands, CSF-1 and IL-34. Inhibiting CSF-1/CSF-1R prevented murine M2 macrophages from differentiating, proliferating, and surviving in one study (169). In addition, blockade of the CSF-1/CSF-1R axis with the specific CSF-1R inhibitors PLX3397, BLZ945, and GW2580 directly impacted macrophage viability and differentiation, improving their function as well as antigen presentation ability. Furthermore, CSF-1R inhibitors induced repolarization of macrophages toward the M1 phenotype and thereby boosted the antitumor T-cell response (84, 171). In an animal model of glioblastoma, CSF-1R blockade demonstrated significant potential to reduce tumor growth, suggesting that CSF-1R inhibitors can block TAM-mediated immunosuppression and make tumor cells more susceptible to chemotherapeutics (120). For instance, treatment with PLX3397 prevented the differentiation of myeloid monocytes into TAMs and improved the response of glioblastoma to ionizing radiotherapy, which delayed the recurrence of glioblastoma (152, 172). Authors reported that the number of TAMs and polymorphonuclear MDSCs in the TME were successfully reduced by the co-targeting of CSF-1R and CXCR2 inhibitors. Importantly, in diverse animal models of cancer, this drug combination reduced tumor burdens and inhibited tumor growth (54, 168, 173).

Also, antibodies against CSF-1 and CSF-1R are used to target macrophages by inhibiting their recruitment and depleting and re-educating them. Given promising results in preclinical data, investigators are further evaluating this combinatorial approach in the setting of breast cancer and other solid tumors in ongoing clinical studies (172). Even though CSF-1R inhibition enhances TAMs’ ability to present antigens in animal models of aggressive pancreatic ductal adenocarcinoma, it may cause exhausted phenotypes of cytotoxic T cells, highlighting the importance of combining immune checkpoint inhibitors and CSF-1R inhibitors in treating these tumors (172).

### 8.2 Polarization of M2 TAMs into tumor-suppressive macrophages

Given the fact that protumor macrophages (M2 phenotype) create an immune-resistant TME whereas antitumor macrophages (M1 phenotype) stop or slow down cancer growth and metastasis, potential strategies for cancer therapy include switching M2 macrophages to the M1 phenotype (120, 162, 173, 174). This change in phenotype may be helpful for cancer treatment because M1 macrophages create an immune-vulnerable microenvironment for cancer cells. Additionally, changing the phenotype of M2 macrophages may stop cancer cells from growing and forming metastases (173, 175). Various substances and modalities to change the state of TAMs within the TEM were investigated including T-cell immunoglobulin and mucin domain 3 and 4 blockade and treatment with macrophage receptor with collagenous structure (MARCO) or Toll-like receptor (TLR) agonists (145, 176–178). TIMs are phosphatidylserine receptors mainly expressed on antigen−presenting cells that are involved in the recognition and efferocytosis of apoptotic cells. They are expressed in immune cells such as NK, T, B, and mast cells and participate in multiple aspects of immune regulation but are also abnormally expressed in cancer cells, contributing to immunosuppression (64, 179, 180). Studies demonstrated that blockade of TIMs improved the anticancer effectiveness of T-cell responses in cancer patients and enhanced the immune cells’ stimulatory properties (64, 180). Investigators achieved similar effects by targeting the scavenger receptor MARCO, which reversed the immunosuppressive effects of TAMs and reduced tumor progression in several murine models of solid tumors (181–183).

Also, use of phosphoinositide 3-kinase γ (PI3Kγ) inhibitors such as IPI-549, mammalian target of rapamycin inhibitors, CD40 agonists, TLR agonists, and class IIa histone deacetylase (HDAC) inhibitors helps repolarize TAMs toward the proinflammatory M1 state (171). Specifically, HDAC inhibitors improved the effectiveness of both chemotherapeutic drugs and immune checkpoint inhibitors in breast cancer treatment by inducing M1 polarization of TAMs (81, 184). The phenotype switch toward M1 was also achieved through PI3Kγ suppression in pancreatic ductal adenocarcinoma, a tactic used to modify the TAM phenotype in solid tumors like melanoma, pancreatic cancer, and lung cancer. They also observed that blocking the PI3Kγ/Akt signaling pathway could decrease the recruitment of integrin α4-dependent MDSCs, increase the recruitment of mature DCs, impede macrophage polarization toward the M2 phenotype, and strengthen T-cell anticancer defenses (185). BKM120 and IPI-549 are two highly effective PI3K inhibitors with direct modifying effects on macrophages and anticancer effectiveness alone or in combination with immune checkpoint inhibitors (83, 90).

Use of TLR agonists has also produced positive results in reversing TAM polarization toward the M1 phenotype. For instance, TLR3 stimulation enhanced the production of MHC-II and other co-stimulatory elements on macrophages by activating the IFN-α/β signaling pathway, exhibited M2/M1 polarization-changing properties, and switched M2 macrophages to the M1 phenotype (186). Also, TLR4 and IFN-γ receptors on macrophages are commonly involved in M1 activation. The major signals associated with M1 macrophage polarization are STAT1 and NF-κB. Immunomodulatory compounds such as Lachnum polysaccharide and glycocalyx-mimicking nanoparticles can interact with TLRs, influencing TAMs to release IL-12, exhibit the M1 phenotype, or reverse the M2 phenotype (158, 187). Of note, glycocalyx-mimicking nanoparticles are internalized by TAMs via lectin receptors, stimulating production of IL-12 and inhibiting production of IL-10, arginase 1, and CCL22 to activate macrophages’ antitumor responses (187, 188). This macrophage phenotype reversion was further controlled by suppressing STAT6 and activating NF-κB phosphorylation (187). Furthermore, glycocalyx effectively reduced tumor burdens in *in vivo* studies and had positive synergistic effects when combined with anti-PD-L1 therapy (145, 187).

Additional targeted nanocarriers have demonstrated efficacy by conveying mRNA-encoding transcription factors responsible for M1 polarization to the M2 phenotype (189, 190). In addition, nanoparticle injections prepared with mRNAs expressing IFN-regulatory factor 5 along with IKKβ switched M2 subsets to antitumor M1 macrophages in animal studies of ovarian cancer, melanoma, and glioblastoma (142). Lately, targeted delivery of chlorogenic acid (CHA) encapsulated in mannosylated liposomes can reduce the immune-suppressive effects of the TME on glioblastoma cells by causing TAMs with the M2 phenotype to adopt an M1 state (191). Remodeling of the TAM phenotype was also caused by infusion of IL-12 in hepatocellular carcinoma models. IL-12 injection lowered the expression of STAT3 and c-Myc, which led to induction of the M1 phenotype in macrophages (192). In another study of hepatocellular carcinoma, IL-37 converted M2 TAMs into M1 cells by inhibiting the IL-6/STAT3 signaling pathway (163). Also, studies using ureido tetrahydrocarbazole derivatives confirmed the potent transformation of M2 macrophages to the M1 phenotype to instill antitumor activity both *in vitro* and *in vivo*. According to Pei et al. (193), the ureido tetrahydrocarbazole derivatives were effective at slowing the growth of tumors in tumor-bearing mouse models and had effective results when combined with anti-PD-1 antibodies. Considering all of these data, altering the phenotype of TAMs to become M1 cells appears to be an effective tactic for increasing the sensitivity of tumor cells to both chemotherapeutic drugs and immunotherapies.

### 8.3 Checkpoints for macrophage-induced phagocytosis

Investigators have identified several tumor-phagocytosis-related checkpoints, including the CD47/signal regulatory protein α (SIRPα) axis, the PD-1/PD-L1 axis, the MHC-I/leukocyte immunoglobulin-like receptor subfamily B (LILRB1) axis, and the CD24/SIGLEC10 axis. This was followed by the development of several mAbs or protein fusions directed against these checkpoints, with some of them exhibiting promising effectiveness in ongoing clinical trials.

#### 8.3.1 CD47/SIRPα checkpoint

The first checkpoint to be connected to tumor phagocytosis was CD47/SIRP cross-talk, commonly referred to as the don’t eat me signal (123). CD47 was first described as a membrane protein in healthy red blood cells (123). Previous studies revealed that senescent red blood cells with reduced CD47 expression are swiftly removed by the macrophages residing in the splenic red pulp, liver tissue, or bone marrow erythroblastic island (6, 72, 194–196). However, in normal erythroid cells, CD47 expression prevents clearance by attaching to the macrophage inhibitory receptor SIRPα (128, 176, 197–199). Recent reports pointed to SIRPα as a membrane protein belonging to the immunoglobulin superfamily that is primarily expressed by myeloid cells like macrophages and other DCs (54). The mechanism behind inhibition of phagocytosis by macrophages was further dissected with the discovery that macrophages and SIRPα interact with CD47 expressed on nearby cells, causing the SIRPα cytoplasmic immunoreceptor to phosphorylate its tyrosine-based inhibitory motif. Src homology 1 and 2 phosphatases are subsequently recruited because of this mechanism (200).

Inhibition of phagocytosis results from the downstream signaling cascade’s prevention of myosin-IIA aggregation at the phagocytic synapse (200). As a result, the CD47/SIRPα axis is mainly thought of as a don’t eat me signal that enables CD47-expressing cells to avoid being phagocytosed by macrophages (200). In contrast, cells lacking CD47 are quickly destroyed by wild-type macrophages (201). Thus, most cell types, including erythroblasts, platelets, and hematopoietic stem cells, express CD47 on their surfaces to avoid being phagocytosed by macrophages (200). However, a similar mechanism of elevating the expression of CD47, and thereby inhibiting macrophage phagocytosis, was found in numerous hematological and solid tumors (129, 198, 202–206). These findings demonstrate that CD47/SIRPα cross-talk acts as a protective immunological checkpoint associated with phagocytosis. Furthermore, authors documented a substantial positive connection between high CD47 expression and poor prognosis for cancer (125, 203, 207–209), leading to several approaches aimed at blockade of this signaling axis. CD47-targeting approaches include the anti-CD47 antibodies Hu5F9-G4 (NCT02216409), SRF231 (NCT035123), and IBI188 (NCT03763149) and the anti-SIRPα antibody BI-765063 (NCT03990233). The anti-CD47 mAb Magrolimab is reported to be the first therapeutic drug to target macrophages (54). These findings demonstrate that suppression of CD47/SIRP cross-talk may indeed improve antitumor activity of macrophages and that using this approach in combination with other therapies may further improve results of immunotherapy (127, 128). Furthermore, clinical studies demonstrated the significance of blocking the CD47/SIRP interaction in animals bearing xenograft models with a variety of hematological cancers, such as acute myeloid leukemia, myelodysplastic syndrome, and refractory non-Hodgkin lymphoma (47, 97, 124, 177, 200, 210).

The results of the studies described above demonstrated that anti-CD47 antibodies facilitate tumor-cell detection and phagocytosis by macrophages (211). Furthermore, macrophage removal reversed tumor development following CD47 blockage, demonstrating that macrophages are essential for suppressing the proliferation of cancer cells after CD47 dampening. Targeting cancer cells with CD47 blockage is carried out using four major strategies (54, 126, 127, 208). (1) Direct killing of cancerous cells. Anti-CD47 mAbs cause tumor cells to die via a process unrelated to caspases (212). (2) Macrophage-regulated antibody-dependent cellular phagocytosis. The use of anti-CD47 mAbs reduces CD47/SIRPα cross-talk, thereby causing macrophages to phagocytose tumor cells (213). Furthermore, inhibiting CD47/SIRPα cross-talk causes tumor cells to be phagocytosed by all macrophage populations, particularly M1 and M2c macrophages (214–216). That study also demonstrated that preventing CD47/SIRPα cross-talk causes a variety of polarized macrophages to engulf tumor cells and that this action is necessary for producing FcγRs (217). This suggests that inhibiting CD47 efficiently causes the diverse macrophage population seen in *in vivo* studies to start destroying tumor cells. Enhancement of antigen presentation ability and CD8^+^ T-cell proliferation *in vitro* are primarily caused by increased cancer cell phagocytosis brought on by the interruption of CD47/SIRPα cross-talk. (3) T-cell-induced immunological responses and DC-mediated antigen presentation. Studies demonstrated that anti-CD47 mAbs stimulate DCs to phagocytose tumor cells, which is followed by antigen presentation to CD8^+^ T cells to trigger an anticancer adaptive immune response (217). (4) NK-cell-modulated antibody-dependent cellular cytotoxicity and complement-dependent cytotoxicity. SIRPα is a notable suppressor of NK-cell-modulated cytotoxicity, whereas anti-CD47 mAbs kill cancerous cells via the antibody-dependent cellular cytotoxicity and complement-dependent cytotoxicity pathways (197). Consequently, preventing CD47/SIRPα cross-talk stimulates the innate and adaptive immune responses, resulting in tumor-cell apoptosis.

**Table 2** includes a list of potential targets and the phases of clinical trials of cancer treatments using these targets performed thus far. Also, in several preclinical studies, researchers have investigated potential therapeutic approaches combining anti-CD47 strategies with anti-CD20 strategies for lymphoma, anti-HER2 strategies for breast cancer, and anti-EGFR strategies for colorectal cancer. The results of these studies indicated that the mechanisms of action of these tumor-opsonizing mAbs can be greatly potentiated by anti-CD47 strategies (240–245). Concerns related to CD47 expression in healthy platelets and red blood cells led to the development of antibodies with weaker anti-CD47 properties and selective SIRPα inhibitors. Several anti-CD47 agents, such as TTI-621 (NCT03530683), TTI-622 (NCT02890368), and ALX148 (NCT04675333), have undergone clinical trial evaluation. In addition to the use of immunomodulatory agents, targeting immune checkpoint pathways could constitute an additional approach. A series of bispecific antibodies combining anti-CD47 specificity with anti-PD-L1, -EGFR, -CD19, or -CD20 activity may preserve tumor-specific phagocytosis-stimulating activities while sparing the host cells that do not express the tumor antigen, thus limiting toxicity. As discussed above, M2 TAMs may possess only low capacity for phagocytosis or the ability to present antigens to cytotoxic T lymphocytes, and thus showing impaired immunological activity. Treatment with antibodies targeting CD47 may be a tactic to help TAMs regain their immunological characteristics. By blocking the connection between CD47 and SIRPα, anti-CD47 antibodies may improve macrophages’ ability to fight tumors (246, 247). Blocking the CD47/SIRPα pathway had promising results in treatment of several solid tumors and hematological cancers such as glioblastoma, lymphoma, and breast cancer and may compel TAMs to phagocytose tumor cells (246, 248–255). Other strategies for harnessing or restoring antitumor properties of macrophages are discussed below.

**Table 2:** Clinical trials of macrophage-targeting therapies for cancer.

Target	Treatment	Phase	Cancer Type	Trial Status	Reference
CSF-1R	Emactuzumab	2	Breast cancer	Closed	(18, 172)
	JNJ-40346527	3	Tenosynovial solid tumors	Closed	(18, 218, 219)
	Cabiralizumab	1	Pancreatic cancer	Open	(18)
	Cabiralizumab + APX005 + nivolumab	1	Melanoma, non-small cell lung cancer, renal cell carcinoma	Active	(18)
	Cabiralizumab (FPA008) + nivolumab (Opdivo)	1/2	Advanced solid tumors	Closed	(18)
	Cabiralizumab (FPA008) + nivolumab (Opdivo)	2	Head and neck tumors	Active	(18)
	Cabiralizumab (FPA008) + nivolumab (Opdivo)	2	Lymphoma	Active	(18)
	Emactuzumab + PD-L1 inhibitor (atezolizumab)	1/2	Advanced solid tumors	Open	(18)
CD47/SIRPα	Hu5F9-G4	1	Solid tumors	Closed	(220)
	Magrolimab	1/2	Acute myeloid leukemia	Closed	(88)
CD40/CD40L	APX005M	1/2	Pancreatic cancer	Closed	(221, 222)
	Selicrelumab	1/2	Melanoma, pancreatic cancer	Open	(49, 223, 224)
CD68	ADG116	1/2	Solid tumors, melanoma, head and neck cancer	Closed	(57, 225–228)
CCR2	PF-04136309	1	Pancreatic cancer	Closed	(57, 98, 146, 149–151)
	CCX872	1/2	Solid tumors	Closed	(57, 98, 146, 149–151)
TLR7/8/9	GSK2831781	1	Solid tumors	Closed	(57, 98, 146, 149–151)
	IMO-2125	1/2	Melanoma, head and neck cancer	Closed	(57, 98, 146, 149–151)
CD206	ANG4043	1	Solid tumors	Closed	(229)
ATM/TTK	AZD1390	1/2	Solid tumors	Closed	(230, 231)
	CFI-402257	1/2	Advanced solid tumors	Closed	(232–234)
CD47	TTI-621	1/2	Solid tumors and hematological cancers	Open	(198)
	AO-176	1	Solid tumors	Closed	(235)
	CC-90002 + nivolumab (Opdivo)	1b/2	Advanced solid tumors	Open	(236)
	Hu5F9-G4 + rituximab	1	Non-Hodgkin lymphoma	Open	(237, 238) (220)
CD115/CSF1R	LY3022855	1	Solid tumors	Closed	(18, 84, 159, 169)
PD-1/PD-L1	Lenvatinib and pembrolizumab	1	Solid tumors	Closed	(239)

#### 8.3.2 Other checkpoint signaling pathways

Additional don’t eat me signals have been identified, such as SIGLEC1 (CD169), the PD-1/PD-L1 axis (161), LILRB, and targeting scavenger antigens. SIGLEC1 (sialoadhesin/CD169) is a membrane protein that binds to sialic acid and mediates cell–cell interactions. CD169 is expressed by a fraction of macrophages that undergo M2 polarization and is upregulated in human cancer cells. As observed with CD47, expression of CD169 correlates with a dismal prognosis in cancer patients (126, 256). Depletion of CD169^+^ TAMs was effective in reducing tumor burdens and metastasis in mouse models of breast cancer, whereas targeting of SIGLEC7 and SIGLEC9 led to a significant reduction in tumor burdens in transgenic mice expressing the human transgenes for SIGLEC7 and SIGLEC9 but lacking expression of the murine homolog Siglec-E that were transplanted with murine B16 and B16-FUT3 lung cancer cells (257). SIGLEC proteins contain immunoreceptor tyrosine-based inhibitory motifs in the cytoplasmic tail, which, through their inhibitory and suppressive activation signals, regulate the functions of several immune cells (51). Another molecule that is frequently overexpressed by diverse tumor types is CD24. Binding of CD24 to SIGLEC10, which is overexpressed by TAMs, leads to phagocytosis inhibition (256). Experimental targeting of SIGLEC10 with mAb against SIGLEC10 restored the phagocytosis properties of macrophages in preclinical models of ovarian cancer (51).

Another approach to stimulating macrophages to regain their antitumor activity may be inhibition of LILRB, a receptor that engages with MHC-I protein (256). Suppression of MHC-I molecules is one of the best-known mechanisms cancer cells use to circumvent recognition by T cells (51, 258). The expression of MHC-I protein by tumor cells was shown to correlate with the level of tumor resistance to anti-CD47 therapy. Of note, like SIGLECs and CD24, LILRB was shown to contain an immunoreceptor tyrosine-based inhibitory motif that exerts an inhibitory activity on immune cells and to be widely expressed by immune cells and enriched in TAMs. Anti-CD47 therapy resistance of tumor cells may be restored by treatment with an LILRB1-blocking antibody. Furthermore, LILRB antagonists such as MK-4830 (NCT03564691), a human mAb directed against LILRB2, in conjunction with IL-4 or macrophage colony-stimulating factor, may alter the ECM composition, limit the recruitment of Tregs to the TME, inhibit the function of MDSCs, and enhance proinflammatory activation and phagocytic activity of macrophages (30, 256, 258, 259). In phase 1 dose-escalation studies in patients with advanced solid tumors, treatment with MK-4830 alone or in combination with anti-PD-1 therapy produced durable responses that correlated with enhanced cytotoxic T-lymphocyte-mediated antitumor immune response. Therapeutic approaches are also targeting LILRB4, and blockade of it had potent activity in reshaping tumor-infiltrating T cells and reversing the M2-suppressive phenotype of TAMs (258).

Other molecules abundantly expressed in TAMs include several types of scavenging receptors. These receptors not only identify specific types of TAMs but also are apparent therapeutic targets with the aim of potentiation of a proinflammatory switch toward the M1 phenotype. Specifically, researchers observed significant correlation between expression of CD163 and progression of several types of solid tumors (139, 167, 260). CD163 enables macrophages to remove erythrocyte debris by binding to haptoglobin. Of note, depletion of CD163^+^ TAMs resulted in tumor regression in a mouse model of anti-PD-1-resistant melanoma (261–264). Furthermore, depletion of CD163^+^ TAMs led to restoration of cytotoxic T-cell and inflammatory monocyte activity, leading to resensitization of tumor cells to anti-PD-1 therapy (265).

Other receptors highly expressed on TAMs, related to the M2 phenotype, are mannose receptor 1 (CD206) and MARCO (181–183). CD206 is a macrophage scavenger receptor that binds to several endogenous ligands in addition to pathogen moieties such as tumor mucins (186, 213). CD206 engages on macrophages maintaining the endocytosis and phagocytosis, and thus immune homeostasis by scavenging unwanted mannoglycoproteins; however, through their interactions with tumor mucins or upon an agonist anti-mannose receptor mAbs, they induced an immunosuppressive phenotype with increased production of cytokines such IL-10 by TAMs (74, 94). Treatment with RP-182 peptide, which binds to CD206/mannose receptor 1 and induces a conformational switch of the receptor, partially depletes CD206^+^ macrophages and reprograms the remaining TAMs into antitumor M1-like effectors with increased inflammatory cytokine production and the ability to phagocytose cancer cells (6, 266). In murine cancer models, RP-182 suppressed tumor growth, extended survival, and synergized with combined immunotherapy (266). Of note, targeting MARCO with mAbs induced mainly an antitumor immune response through reprogramming of TAMs (267).

Immunosuppressive M2-like macrophages also express the receptor Clever-1 (stabilin-1), an adhesion and scavenger receptor. Clever-1 binds to several ligands, primarily lipoproteins and carbohydrates, mediating endocytosis of scavenged material and its delivery to the endosomal compartment, ultimately resulting in suppression of macrophages and impaired activation of Th1 lymphocytes (268, 269). Antibody blockade of Clever-1 with FP-1305 caused a phenotypic switch in TAMs from immunosuppressive to proinflammatory and activation of T-cell responses and delayed tumor growth in preclinical studies (269–272). These preclinical results led to a phase 1 trial to determine the safety and preliminary effectiveness of FP-1305, a humanized anti-Clever-1 antibody administered to heavily pretreated patients with metastatic solid tumors (269). Encouraging results of this trial indicated a proinflammatory switch of monocytes, enhanced capability of macrophages to cross-present scavenged antigens, and activation of T cells (270). TAMs also express PD-1, which inhibits phagocytosis and tumor immunity, impairing the PD-1/PD-L1 axis in macrophages. Of note, PD-L1 expression in cancer cells may concomitantly enable evasion from not only T-cell cytotoxicity but also macrophage-mediated phagocytosis (273–275). Therefore, blockade of the PD-1/PD-L1 axis may enhance an antitumor immunity of both adaptive and innate mechanisms.

Of note, the receptors PD-1, LILRB1, and SIRPα all contain an immunoreceptor tyrosine-based inhibitory motif domain, which may be instrumental for downstream signals that inhibit phagocytosis (258, 259, 274, 276, 277). Based on this, in studies aimed at monitoring response in patients with cancer undergoing treatment with immune checkpoint inhibitors, researchers should consider the myeloid compartment as a potential target and predictive biomarker (104, 160). TAMs were also shown to upregulate triggering receptor expressed on myeloid cells 2 (104, 237, 274, 278). This protein scavenges large molecules like lipoproteins and phospholipids as well as cell debris. Targeting of triggering receptor expressed on myeloid cells^+^ TAMs led to restricted tumor growth and resensitization to anti-PD-1 therapy. Investigators recently evaluated PY414, a humanized mAb targeting triggering receptor expressed on myeloid cells 2^+^ macrophages, in a phase 1 clinical trial in patients with advanced solid tumors (NCT04691375) (237, 278). Finally, another ligand strongly upregulated in M2 macrophages and expressed in TAMs is P-selectin glycoprotein ligand-1 (279). This protein has high affinity for VISTA (B7-H5 and PD-1H) and selectins, and upon activation, it contributes to T-cell dysfunction in cancer patients (280). Targeting of P-selectin glycoprotein ligand-1 should be a subject of further investigation.

### 8.4 Targeting epigenetic and metabolic changes in TAMs

Therapy resistance may be a consequence of metabolic rewiring in both tumor cells and cellular immune compartment of TME. Downstream metabolic rewiring of macrophage function following polarization changes involves complex changes in amino acid, lipid, and iron metabolism (19, 69, 134, 196, 281, 282). This complex series of events provides potential targets to rewire macrophage function at the metabolic level. One of the promising approaches to harnessing the antitumor potential of macrophages is epigenetic regulation by class IIa HDACs. TMP195, a selective class IIa HDAC inhibitor, exhibited the ability to effectively modify the transcription profile of macrophages, resulting in macrophage-mediated reduction of tumor growth in a breast cancer model (81, 184). Another HDAC inhibitor, tefinostat (CHR-2845), is cleaved to an active acid form CHR-2847 via nonspecific esterase liver carboxylesterase 1, an enzyme selectively present only in monocytoid-lineage cells and some hepatocytes. Because of this feature, tefinostat has been successfully tested in a phase 1 clinical trial in patients with advanced hematological cancers such as myelodysplastic syndrome and chronic myeloid leukemia (NCT00820508). Also, carboxylesterase 1 may be used as an elegant tool for developing drugs with macrophage-selective targeting features.

Also, hypoxia and acidosis (**Figures 3**, **4**) play a crucial role in the TME and can modulate the function of TAMs. For instance, the oxygen demand of initially fast-proliferating tumor cells may enhance the hypoxic gradient across tumor tissue, forcing both tumor cells and immune cells to adapt to new conditions. Metabolic wiring may therefore promote nonoxidative pathways of energy generation, which leads to increased tumor acidification. Hypoxia can trigger the expression of genes like TNF-α, IL-18, and H1F-1 in TAMs, which may cause inflammation, angiogenesis, and tumor growth. Both hypoxia and acidification were shown to promote polarization of macrophages toward the M2 phenotype and therefore may consolidate the protumorigenic milieu. Therapeutic interventions impeding hypoxia or hypoxia-inducible changes such as blockade of HIF-2 with belzutifan in renal cell cancer cells and use of hypoxia-activated prodrugs may constitute an important backbone of macrophage-targeted therapies (111, 116, 283).

Another opportunity for targeting TAMs and antitumor therapy may be blockade of other metabolic pathways, such as OXPHOS. Given the fact that M2 macrophages and some subsets of hematological cancers and stem cell populations in solid tumors rely more on OXPHOS than other metabolic pathways for biosynthetic and bioenergetic demands, selective blockade of OXPHOS (281, 284) may be synergistic together with anti-CD47 therapy, in both achieving direct eradication of OXPHOS-dependent tumor cells and reshaping the TME through elimination of protumorigenic, OXPHOS-dependent M2 macrophages. Along this line, treatment with the respiratory complex I inhibitor metformin, an antidiabetic agent, reduced the density of TAMs, remodulated their function in the TME, and increased their phagocytic function, and its antitumor efficacy has been tested in several clinical trials for the treatment of diverse types of cancer (260).

M2 TAMs are often characterized by increased consumption of glutamine, which is essential for biosynthetic processes and redox balance. Thus, combined small-molecule inhibitors such as CB-839 and DON downstream from glutamine receptors may be therapeutic options for modulation of myelosuppressive cells (285). Another amino acid of great interest in macrophage targeting is tryptophan. Increased consumption of tryptophan by TAMs owing to elevated expression of the enzyme indoleamine 2,3-dioxygenase 1 results in reduced tryptophan access for T cells and accumulation of kynurenine, leading to severe impairment of cytotoxic T-cell function, and inhibits T lymphocytes division and favors T-cell differentiation toward Treg generation (286). Whereas some results of ongoing clinical trials testing indoleamine 2,3-dioxygenase 1 inhibitors alone or combined with other agents such as pembrolizumab have been negative, results for other combinations using anti-PD-1 agents are pending (190, 239, 287–292).

Another metabolic vulnerability of TAMs is lipid metabolism. Researchers showed that TAMs possess a defective mechanism of lipid utilization that is most likely linked to activation of immunosuppressive pathways and mediated by the oxysterol receptor and transcription factor LXR (293). Strategies targeting LXR such as exposure to LXR agonists have induced anti-inflammatory actions and reduced the pool of macrophages in affected lesions. Another group of lipid derivatives, prostaglandins, particularly tumor-derived prostaglandin E2, blocked early activation of NK cells and inflammatory activation of myeloid cells, consolidating the immune-suppressive phenotypes of the TME (77). Furthermore, altered prostaglandin pathways have negatively impacted the effectiveness of ICB, which could be reversed and enhanced by use of prostaglandin G/H synthase 2 (cyclooxygenase-2) inhibitors or antagonists of the prostaglandin E2 receptors EP1 and EP2 (51, 77).

Another metabolic factor facilitating cancer therapy resistance is acidosis, particularly lactic acidosis. Lactic acid produced by tumor cells as a by-product of glycolysis can lead to upregulation of the CD206 and CD163 genes in TAMs, which is linked to M2 polarization and immunosuppression. Lactate functionally polarizes macrophages toward an M2-like phenotype and leads to elevated expression of arginase 1 (294) (**Figure 4**), suggesting that targeting glycolysis in general or lactate flux inhibition in particular positively influences TAM polarization and activity. Other synergistic effects of metabolic interventions that may impair acidosis-driven TAM polarization toward the M2 phenotype or reuse of lactate in solid tumors can be achieved via selective blockade of lactate transporters such as monocarboxylate transporters 1–4 (MCT1–4) (295) or inhibition of glycolysis pathways, for which novel MCT receptor family inhibitors warrant further investigation on their efficacy to inhibit lactate release into TME. Recently, authors discussed the role of metabolic reprogramming in the context of ICB failure. Therefore, combined metabolic and immune interventions may be novel, promising solutions for counteracting the ICB resistance (282).

Moreover, hypoxia and acidosis can negatively impact the secretion of cytokines such as IL-10 by TAMs, which can hinder the immune response and promote tumor survival. Overall, the effects of hypoxia and acidosis on TAMs are multifaceted and rely on specific genes and cytokines. Comprehending these effects can provide valuable insight into the mechanisms of tumor immune evasion and may open doors for developing innovative immunotherapeutic strategies for cancer as summarized in **Figures 3**–**5**.

### 8.5 Chimeric antigen receptor macrophages

As described above, TAMs can make up almost half of the cellular mass of a tumor (31, 46). However, the TAM pool undergoes continuous restructuring through the recruitment of new circulating monocytes (35, 74, 82). Compared with hematological cancers, which are effectively targeted in many cases by chimeric antigen receptor (CAR) T cells, treatment of solid tumors with CAR-T therapy owing to vascular remodeling, hypoxia, and acidosis is often less effective (7, 57, 80, 120, 129, 177). Given the constant trafficking of monocytes into tumors, macrophage-based cell therapies may constitute a feasible alternative to overcome obstacles to treat solid tumors, associated with the use of CAR T cells (15, 57, 80, 120, 129, 177). Thus, engineering macrophages to deliver cytokines or nanoparticles to the TME or equipping them with specific receptors may be a promising therapeutic approach. Researchers have looked at using monocytes replenished with drug-loaded nanoparticles or capable of delivering IFN-α to a tumor site and consequently activating an immune response in preclinical studies. They subjected hematopoietic progenitors under the Tie2 promoter to IFNA1 gene transduction. Tie2-expressing monocytes, which have a high level of tumor-homing ability, successfully migrated to tumors and delivered IFN-α to the TME, triggering the activation of immune cells and inhibiting tumor growth and angiogenesis (108, 145, 177).

Furthermore, studies using soft particles as “backpacks” containing cytokines demonstrated that backpacks were stuck on macrophage surfaces, causing acquisition of the M1 phenotype regardless of the presence of an immunosuppressive TME and leading to significant reduction of tumor growth and metastatic burdens (296). Another approach to modify macrophages was genetic engineering of myeloid cells to express IL-12. This approach elicited a type 1 immune response and reduced metastasis and primary tumor growth (51, 297).

Although transducing human macrophages remains a challenge in developing mononuclear-phagocyte-based cellular therapies for cancer, investigators recently developed several innovative therapies to overcome this obstacle. New-generation CAR macrophages armed with receptors recognizing carcinoembryonic antigen-related cell adhesion molecule 5, CD19, CD22, HER2, and CD5 to improve macrophage’s detection and clearance in patients with hematological malignancies and solid tumors are undergoing preclinical and clinical evaluation (80, 105–108, 145, 177). Despite first promising results, there is still an unmet need to enhance CAR-macrophage-mediated phagocytosis of tumor cells and to provide a solution on maintaining the M1 shape and functions in a stable way regardless of tumor environment together with improving the trafficking of CAR-M into primary and metastatic tumors that should be further investigated.

## 9 Future recommendations and conclusions

The cross-talk between macrophages and tumor cells plays a critical role in cancer progression and represents a promising target for cancer treatment. However, further research is needed to understand the molecular mechanisms underlying this complex cell–cell communication. Modulation of macrophage polarization, blockade of signaling pathways, and disruption of physical interactions among macrophages and tumor cells are strategies developed to target this cross-talk. The preclinical and clinical evidence supporting the effectiveness of these strategies is promising. To provide better, more targeted, safe, effective cell-specific therapeutic strategies, more research is warranted to fully understand the molecular mechanisms of these processes. In fact, combinatorial therapies that target multiple aspects of the macrophage–tumor cell cross-talk may be more effective than single-agent therapies, such as modulation of macrophage polarization, blockade of signaling pathways, and disruption of physical interactions. In addition, development of imaging techniques together with *in vitro* and *in vivo* studies of potential biomarkers to monitor the presence, activation state, and function of macrophages in tumors will aid in selecting patients who could benefit from macrophage-targeted therapies. Preclinical and clinical studies of TAMs in cancer should focus on the specific roles of macrophages in different types of tumors to identify the most promising tumor-type-specific targets for therapy. Development of *in vitro* and *in vivo* models that accurately recapitulate the complex interactions between macrophages and tumor cells will be essential to further our understanding of this cross-talk and test new therapeutic strategies. Finally, further study is needed to understand the potential side effects and toxicity of macrophage-targeted therapy, mainly when combined with other cancer treatments. Careful monitoring of potential side and toxic effects therefore is essential when developing macrophage-targeted therapies, particularly in combination with other cancer treatments.

## Author contributions

MA: Visualization, Writing – original draft, Writing – review & editing. AK: Visualization, Writing – original draft, Writing – review & editing. FK: Visualization, Writing – original draft, Writing – review & editing. MK: Visualization, Writing – original draft, Writing – review & editing. EM: Visualization, Writing – original draft, Writing – review & editing. MN: Visualization, Writing – original draft, Writing – review & editing. NB: Visualization, Writing – original draft, Writing – review & editing, Funding acquisition, Supervision.
